# Enhanced Magneto-Optical Kerr Effect in Nano-Magnetic Metamaterials with Near-Perfect Coherent Absorption

**DOI:** 10.1021/acsnano.6c15462

**Published:** 2026-10-21

**Authors:** Xiaofei Xiao, Tingjun Zheng, Alex Vanstone, Daniel S. Bromley, Holly Holder, Kilian D. Stenning, Jack C. Gartside, Will R. Branford, Rupert F. Oulton

**Affiliations:** † Blackett Laboratory, 4615 Imperial College London, London SW7 2AZ, United Kingdom

**Keywords:** metamaterials, optical absorption enhancement, magneto-optical Kerr effect, nanomagnetism, all-optical magnetic switching, magnetic light absorption

## Abstract

The ability to control magnetic systems without external magnetic fields has attracted attention recently, with all-optical magnetic switching showing increasing promise. Gaining fine control over light-matter interactions in magnetic nanostructures is essential for advancing opto-magnetic technologies but remains challenging because strong optical coupling must be achieved within ultrathin magnetic structures at the target wavelength. Here, we show that coherent optical interference can increase this interaction in patterned magnetic metasurfaces. Arrays of permalloy nanomagnets are placed on a thin dielectric spacer above a reflective back-plane, forming a magnetic metasurface whose reflected wavefront is defined by the nanomagnet geometry, fill factor, and spacer thickness. Destructive interference between the metasurface and back-plane reflections yields near-perfect absorption, exceeding 95% for circular nanomagnet arrays in both simulation and experiment. The absorbed power is dissipated almost entirely within the nanomagnets, with only a few percent lost in the metallic back-plane. Enhanced absorption is also accompanied by a stronger magneto-optical Kerr effect, driven by both increased light–nanomagnet interaction and reduced reflected background. These results provide a general approach for achieving high optical absorption and strong magneto-optical response in nanoscale magnetic systems, supporting advances in optically addressable spintronics, magnetic sensing, and all-optical magnetic control.

## Graphical abstract

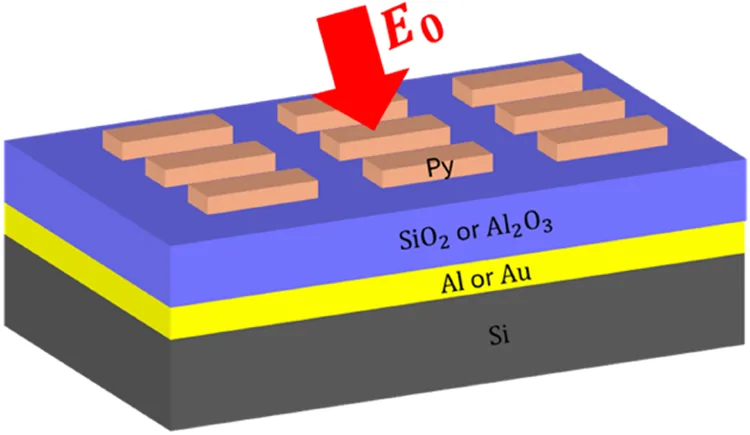

## Introduction

The use of light to manipulate magnetic states has come to prominence in recent years through investigations of optical demagnetization,^1–3^ all-optical switching,^4–8^ optically addressed magnetic sensors,^9^ and plasmon-assisted thermal control of nanomagnets,^10,11^ with applications in spintronic computing and data storage. Enhancing light’s interaction with magnetic materials has thus become important for a large range of emerging technologies. The most widely used magnetic materials are metallic, and their loss is strongly frequency dependent;^12^ many ferromagnetic alloys absorb strongly in particular spectral windows. The difficulty is achieving strong interaction at the target wavelength. Magnetic device designs require layer thicknesses from a few nanometers to tens of nanometers, which substantially limits the optical interaction volume, especially when patterned into nanostructures. This has motivated interest in the plasmonics of magnetic metals,^13,14^ however, the same large imaginary permittivity that provides absorption also damps the resonance, limiting plasmonic enhancement. Indeed, hybrid plasmonic systems that combine magnetic and noble metals have also been considered.^15^ One strategy to overcome this limitation is to use thin-film interference, where multilayer stacks of dielectrics and metals^16,17^ manipulate light waves to control their reflection, transmission and absorption. For example, large enhancements of the magneto-optical Kerr effect (MOKE) were reported in planar stacks of dielectric and magnetic layers by enhancing absorption.^18^ Enhanced absorption has also recently been shown to be important in all-optical switching of states in magnetic metamaterials comprising dense arrays of strongly coupled nanomagnets (artificial spin ice - ASI) using low power continuous wave lasers.^7^

Achieving high absorption in thin materials is essential for a variety of technologies throughout the electromagnetic spectrum,^7,15,19–24^ including photovoltaic and photochemical cells, photodetectors, optical filters, stealth technology, and thermal light sources. Conventional routes to increasing interaction and absorption rely on techniques, such as thin-film interference, plasmonics and metamaterials.^25–32^ Researchers have also explored the use of thin but absorptive materials (including semiconductors and metals) as nanometer-thick optical coatings^20,33^ to control reflection and transmission across the ultraviolet, visible, infrared, and terahertz spectral ranges.^16,34–37^ While thin-film interference has been shown to enhance magneto-optical responses in continuous planar magnetic films,^18^ its implementation in lithographically patterned metallic nanomagnets creates a direct coupling between optical and magnetic design: the nanomagnet geometry and magnetic-material fill factor determine both the optical response and the magnetic functionality of the structure. Existing routes to enhancing MOKE responses include interference-based cavities and multilayer structures,^38^ plasmonic and magneto-plasmonic nanostructures,^39–42^ all-dielectric metasurfaces and photonic resonators exploiting Mie, guided, or quasi-bound states in the continuum resonances,^43,44^ intrinsic and interfacial material engineering,^45^ and measurement-side amplification schemes such as weak-value amplification.^46^ A detailed comparison of these approaches is provided in the Supporting Information.

In this report, we demonstrate interference-assisted coherent absorption in lithographically patterned permalloy nanomagnet arrays placed above a thin dielectric spacer and a reflective metallic back-plane. The nanomagnet geometry and magnetic-material fill factor control the optical response of the metasurface, enabling destructive interference of the reflected waves and near-perfect absorption exceeding 95%, with only a few percent of the incident power dissipated in the metallic back-plane. We further show that the enhanced light–nanomagnet interaction is accompanied by an increased longitudinal MOKE response from in-plane-magnetized nanobars. The approach therefore provides a lithographically tunable route to controlling optical absorption and magneto-optical response directly through the geometry of functional magnetic nanostructures.

## Results and Discussion

### System Description

The permalloy (Py) metasurface interference system consists of four layers, as shown in Figure 1a. Light is incident from the top, considered air, onto the metasurface layer of the permalloy (Ni_81_Fe_19_), whose structure consists of circular disks or bars. As shown in Figure 1b, this layer can be viewed as an effective medium of zero thickness, where its true thickness, fill factor, and nanomagnet shape determine the reflection coefficients from Region 1 (2) to Region 1 (2), *r̃*_1_ = |*r̃*_1_|*e* ^*iψ*_*r*1_^ and *r̃*_2_ = |*r̃*_2_|*e* ^*iψ*_*r*2_^, and the transmission coefficients from Region 1 (2) to Region 2 (1), *t̃*_1_ = |*t̃*_1_|*e* ^*iψ*_*t*1_^ and *t̃*_2_ = |*t̃*_2_|*e* ^*iψ*_*t*2_^. Reflection and transmission from the metasurface are assumed to be specular with no diffraction orders, since the nanomagnets and their period are both subwavelength in scale. The permalloy metasurface sits on a dielectric layer of variable thickness, *l*, which controls the phase change, Φ = *k*_*zl*_, of light passing through it, where *k*_*z*_ is the *z*-component of the wavevector. The final layer is the metal back-plane (either aluminum or gold, in our case), which should be highly reflective, *r̃*_*b*_ = |*r̃*_*b*_|*e* ^*iψ*_*rb*_^, across the desired operation wavelengths; in this case, the visible and near-infrared. Note that the reflection phase of the metal back-plane ψ_*rb*_ is not simply π due to the finite conductivity of real metals and their highly dispersive permittivity in the frequency region of interest.^16^ The near-perfect absorption in this system originates from Fabry–Pérot-type interference induced by multiple reflections within the multilayer structure, enabling coherent absorption. The total reflection, *r̃*_*t*_, accounting for multiple reflections within the system is,

1 $${\mathrm{r̃}}_{t}={\mathrm{r̃}}_{1}+{\mathrm{t̃}}_{1}{\mathrm{r̃}}_{b}{\mathrm{t̃}}_{2}e^{2i\Phi }\sum_{n=0}^{n_{\max}\to \infty }{\left({\mathrm{r̃}}_{2}{\mathrm{r̃}}_{b}e^{2i\Phi }\right)}^{n}={\mathrm{r̃}}_{1}+\frac{{\mathrm{t̃}}_{1}{\mathrm{t̃}}_{2}{\mathrm{r̃}}_{b}e^{2i\Phi }}{1-{\mathrm{r̃}}_{2}{\mathrm{r̃}}_{b}e^{2i\Phi }}$$

**Figure 1:**
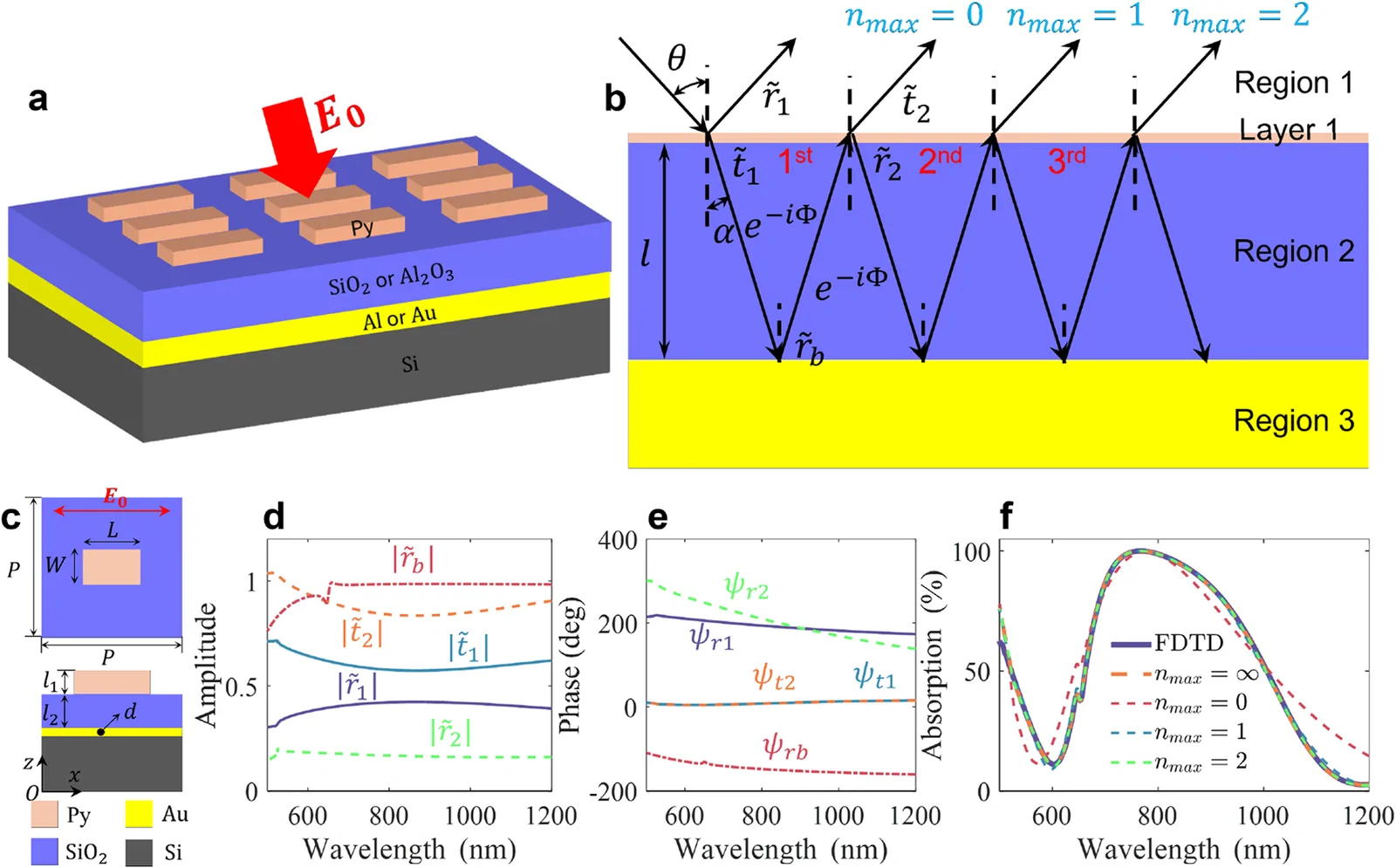
Schematic of structured nanometer optical coatings. (**a**), Schematic illustration of isolated nanostructured lossy material (Py nanomagnets) patterned on a metallic film (Al or Au), deposited on a thick substrate (Si) with a dielectric coating (SiO_2_ or Al_2_O_3_). (**b**), Schematic of a simplified four-layer structure under oblique incidence. An incident beam from Region 1 (air) is partially reflected at Layer 1 (Py), which is assumed to have negligible thickness, and partially transmitted into Region 2, which has a finite thickness *l*. The transmitted wave propagates through Region 2 until it reaches Region 3 (the metallic ground plane), accumulating a complex propagation phase. Upon reflection at the ground plane and acquisition of an additional propagation phase, the wave undergoes partial reflection at the interface between Region 2 and Layer 1, and partial transmission back into Region 1 via Layer 1. This process repeats indefinitely, with the light gradually diminishing. (**c**), Schematic of the unit cell in an exemplary nanomagnet system. The rectangular permalloy nanomagnet arrays are arranged in a square lattice. The SiO_2_ and the gold mirror serve as a cavity system. Amplitude (**d**) and phase (**e**) of the reflection and transmission coefficients in the theory for an illustrative system: The designed nanomagnet system has dimensions: *l*_1_ = 20 nm, *l*_2_ = 375 nm, *L* = 250 nm, *W* = 100 nm, *d* = 250 nm, and *P* = 360 nm. The incident light is *x*-polarized. (**f**), Absorption spectra calculated using the interference model (dashed lines) and full FDTD simulation (solid line). The dashed curves correspond to truncating the summation in eq 1 at *n*_max_ = 0, 1, 2, and ∞.

The total absorption of the system can be calculated by *A*_*t*_(ω) = 1 – *R*_*t*_(ω) – *T*_*t*_(ω) ≈ 1 – |*r*_*t*_|^2^, since *T*_*t*_(ω) = 0 for a thick, highly reflective back-plane. For the aluminum back-plane, the ground plane absorbs no more than a few percent of the incident light across the wavelength range studied here (see Supporting Information, Figure S1), while the rest is absorbed by the magnetic metasurface; the same holds for gold above its interband edge. Our goal is thus to suppress the reflection, *R*_*t*_, thereby enhancing absorption by the metasurface.

To analyze the physics, consider the exemplary nanomagnet system shown in Figure 1c. We first computed the reflection and transmission coefficients with their respective phases for the magnetic metasurface alone for normal incidence as shown in Figure 1d and e. Using these effective parameters, the normal-incidence absorption of the complete structure with metal back-plane was then calculated using eq 1, with the multiple-reflection summation truncated at *n*_max_ = 0, 1, 2, and ∞, as shown in Figure 1f. The absorption of the complete optical coating system was also rigorously simulated as a three-dimensional (3D) coupled magnetic metamaterial multilayer structure using the finite-difference time-domain (FDTD) method (Lumerical FDTD). The computed absorption obtained from the simplified interference model agrees closely with the rigorous numerical simulations. The agreement deteriorates only marginally as the summation is truncated at lower orders, with *n*_max_ = 2 already yielding negligible deviation from the full result. Even for *n*_max_ = 0, the interference model remains sufficiently accurate and simplifies to,

2 $${\mathrm{r̃}}_{t}\approx {\mathrm{r̃}}_{1}+{\mathrm{t̃}}_{1}{\mathrm{r̃}}_{b}{\mathrm{t̃}}_{2}e^{2i\Phi }\approx \left|{\mathrm{t̃}}_{1}\right|\left|{\mathrm{t̃}}_{2}\right|\left|{\mathrm{r̃}}_{b}\right|e^{i\left(\psi_{\mathrm{rb}}+2\Phi \right)}-\left|{\mathrm{r̃}}_{1}\right|$$

where we have applied the values ψ_*r*1_ ≈ π, ψ_*t*1_ = ψ_*t*2_ ≈ 0, from the computed reflection and transmission coefficients of the magnetic layers, shown in Figure 1d and e. To enhance absorption, destructive interference of the reflected waves is necessary, where the SiO_2_ layer thickness should be chosen such that ψ_*rb*_ + 2Φ ≈ 2*mπ* for an integer *m*. Near-perfect absorption can then be achieved when |*t̃*_1_||*t̃*_2_||*r̃*_*b*_| = |*r̃*_1_|, which can be controlled by varying the permalloy nanostructure shape as well as the fill factor.

In the remainder of the paper, our quantitative predictions use FDTD simulations to calculate the optical absorption of the system. In our experiments, we study the absorption by the permalloy nanomagnet metasurface using Fourier transform infrared (FTIR) spectroscopy, while we study the magneto-optical interaction using longitudinal MOKE measurements. For normal-incidence simulations and measurements, the polarization is defined with respect to the Cartesian coordinates, where *x*- and *y*-polarized light correspond to electric fields oriented along the *x*- and *y*-axes, respectively. For oblique-incidence MOKE measurements, the polarization is defined relative to the plane of incidence. Since the measurements were performed in the *xz*-plane, *s*-polarization corresponds to an electric field along the *y*-direction, while *p*-polarization corresponds to an electric field within the *xz*-plane.

### Tuning Absorption via Reflection Phase

We first explore the enhancement of optical absorption by tuning the thickness *l* of the dielectric spacer layer. Figure 2a schematically depicts the magnetic metasurface unit cell and layer structure. The metasurface consists of permalloy nanobars arranged in a square ASI geometry, where half of the bars are oriented along the *x*-axis and the other half along the *y*-axis. A representative scanning electron microscope (SEM) image of the fabricated structures is shown in Figure 2b, demonstrating high-quality patterning over large areas. The dielectric spacer thickness is varied to modulate the optical cavity properties and achieve spectral tuning of the absorption.

**Figure 2:**
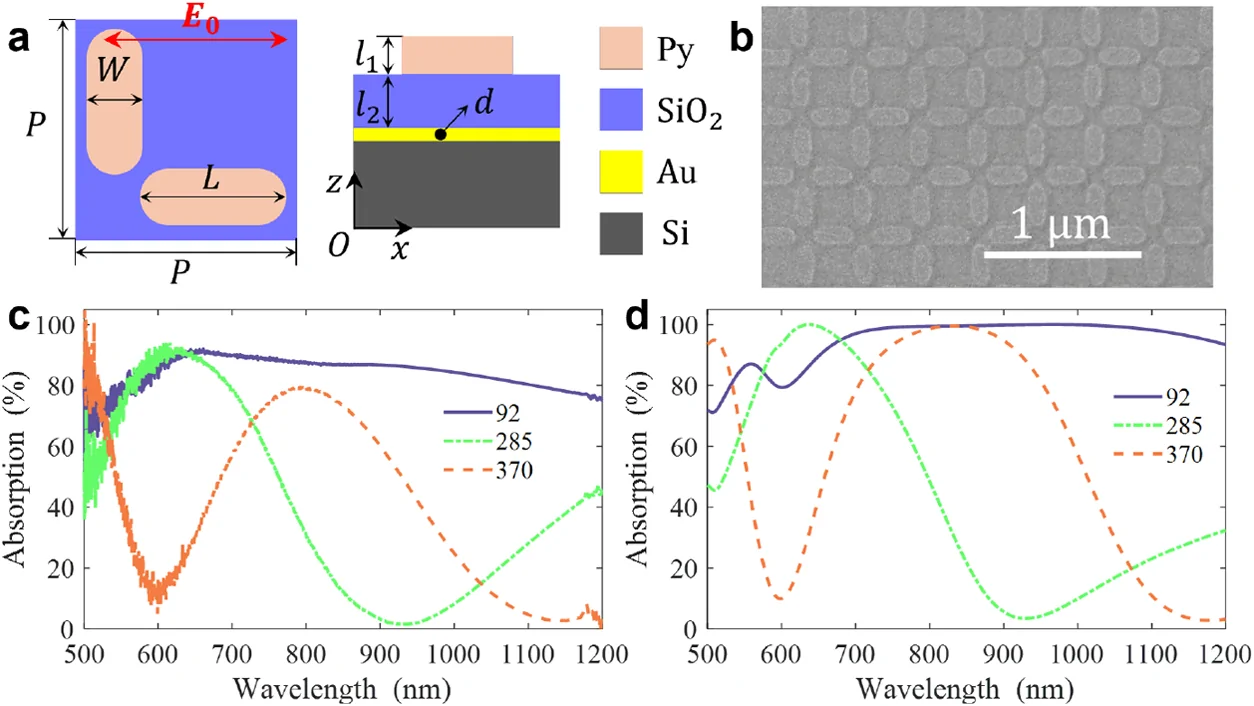
Thickness-controlled absorption of the structured nanometer optical coatings. (**a**), Schematic of the nanomagnet array structures with varying cavity thickness (*l*_2_). (**b**), SEM image of the fabricated periodic nanomagnet structures. The scale bar represents 1 μm. Measured (**c**) and simulated (**d**) absorption spectra of nanomagnet arrays for varying SiO_2_ thickness (*l*_2_). The designed nanomagnet system has dimensions: *l*_1_ = 20 nm, *L* = 250 nm, *W* = 110 nm, *d* = 250 nm, and *P* = 360 nm. The incident light is x-polarized.

FTIR measurements were performed at near-normal incidence over the 500–1200 nm spectral range for varying silica thicknesses, as shown in Figure 2c. The corresponding FDTD simulations, shown in Figure 2d, reproduce the measured absorption spectra well. Minor discrepancies are attributed to fabrication tolerances, such as edge roughness, grain boundaries, and slight variations in silica thickness. The measured absorption of this ASI nanobar array peaks at >80% and can be spectrally tuned across a wide range of frequencies by modifying the spacer thickness, while simulations for the same structure give >95%. Nonetheless, the correspondence of experiment and theory is close with spectral features clearly correlated. Notably, an octave-spanning broadband absorption is obtained for the lowest order of spacer thickness.

The 500–1200 nm window is set by the materials and by our instrument. Gold loses its near-unity reflectivity below its interband edge near 515 nm (see Supporting Information, Figure S3a,b), degrading the interference condition for the Au back-plane of Figure 2; aluminum retains high reflectivity further into the blue, so for the remaining samples the short-wavelength bound is instead set by the usable range of our FTIR system. At long wavelengths the round-trip phase detunes from the value designed at 633 nm. Neither bound is fundamental, since the condition can be re-established elsewhere by scaling *l*_2_ ∝ λ and choosing a mirror that remains reflective there.

### Tuning Absorption via Magnetic Metasurface Fill-Factor

We now examine absorption control via the metasurface reflection and transmission using both the shape and fill factor of magnetic nanostructures. In ASI structures, the optical coupling between perpendicular permalloy bars complicates our analysis, so we use new metasurfaces with single nanostructure bases. Figure 3 presents the two sets of nanomagnet arrays used. Figure 3a and b show circular and rectangular nanomagnet unit cells, respectively, with the arrows indicating the incident light polarization used in each case. Absorption tuning is achieved by adjusting the nanomagnet dimensions; the diameter *D* in the circular case, and the length *L* and width *W* in the rectangular case. These parameters thus also control the fill factor. Here, the fill factor *f* denotes the projected fraction of the unit-cell area occupied by permalloy. For the square arrays used in this work, *f* = *πD* ^2^/(4*P* ^2^) for circular nanomagnets and *f* = *LW*/*P* ^2^ for rectangular nanobars, where *P* is the lattice period. Permalloy is employed as the magnetic medium, while SiO_2_ serves as the lossless dielectric spacer layer, fixed at a thickness of 92 nm in this study to provide broad absorption as observed in Figure 2. Figure 3c shows the layers of the design. Representative SEM images of the fabricated circular and rectangular arrays are shown in Figure 3d and e, respectively, highlighting the high fidelity of the nanofabrication.

**Figure 3:**
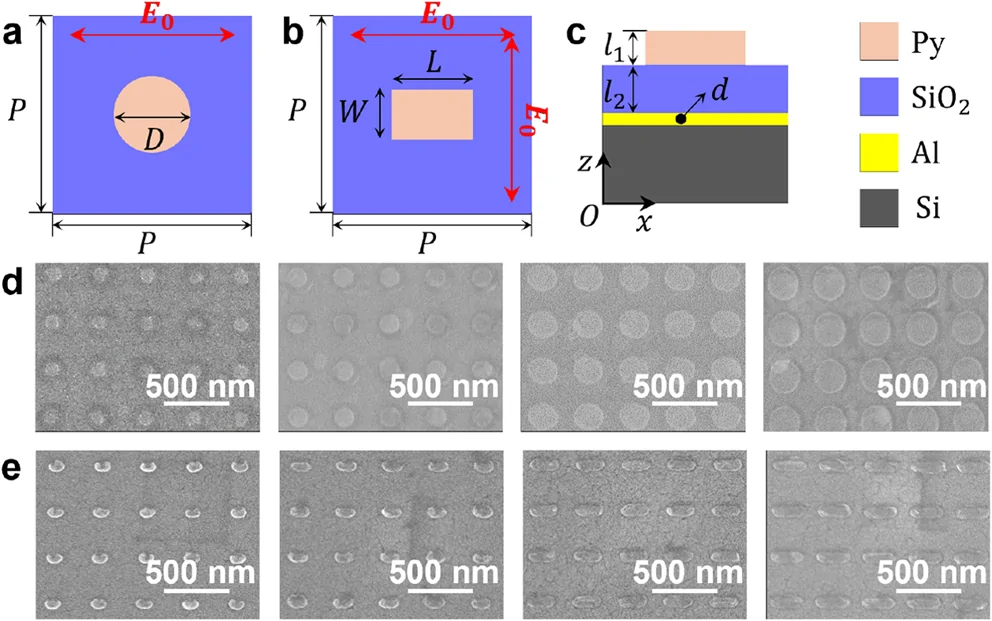
Structure geometry and fabricated samples for fill-factor-controlled absorption. (**a**, **b**) Top view and (**c**) side view of the unit cell of the nanomagnet system. The (**a**) circular or (**b**) rectangular permalloy nanomagnet arrays are arranged in a square lattice. The SiO_2_ and the aluminum mirror serve as a cavity system. The absorption response can be controlled by varying the size of the permalloy nanomagnet, the thickness of the SiO_2_ (*l*_2_) and the nanomagnets (*l*_1_). The nominal geometric parameters are *l*_1_ = 20 nm, *l*_2_ = 92 nm, *W* = 100 nm, and *d* = 250 nm. The period of the square lattice is fixed to *P* = 360 nm. All structures were fabricated on a silicon substrate. Linearly polarized light at normal incidence illuminates the system from the air side. For circular nanomagnets, we use *x*-polarized light, while for rectangular nanomagnets, we apply both *x*- and *y*-polarized light. SEM images of the fabricated (**d**) circular (of diameter *D*) and (**e**) rectangular (of length *L* and width *W*) nanomagnets with various sizes. The scale bars represent 500 nm.

We fabricated six samples with circular nanomagnets of varying nominal diameters and characterized their optical response using FTIR spectroscopy under linearly polarized light (*x*-polarization). The simulated and experimentally measured absorption spectra are presented in Figure 4a and b, respectively. The two sets of results show excellent agreement, validating our simple and intuitive interference model. Notably, in the simulations, the absorption maximum exhibits a systematic red-shift with increasing nanomagnet diameter, indicating a broad, geometry-dependent optical response of the lossy permalloy nanostructure. This condition corresponds to |*t̃*_1_||*t̃*_2_||*r̃*_*b*_| ≈ |*r̃*_1_|, where the reflection is strongly suppressed. For the circular-disk metasurface, the absorption exceeds 95% at *D* = 205 nm in both simulation and experiment, as shown in Figure 4b, corresponding to a fill factor of 25.5%. This shows that near-perfect destructive interference of the reflection may be achieved, and thus near perfect absorption in the permalloy nanostructures.

**Figure 4:**
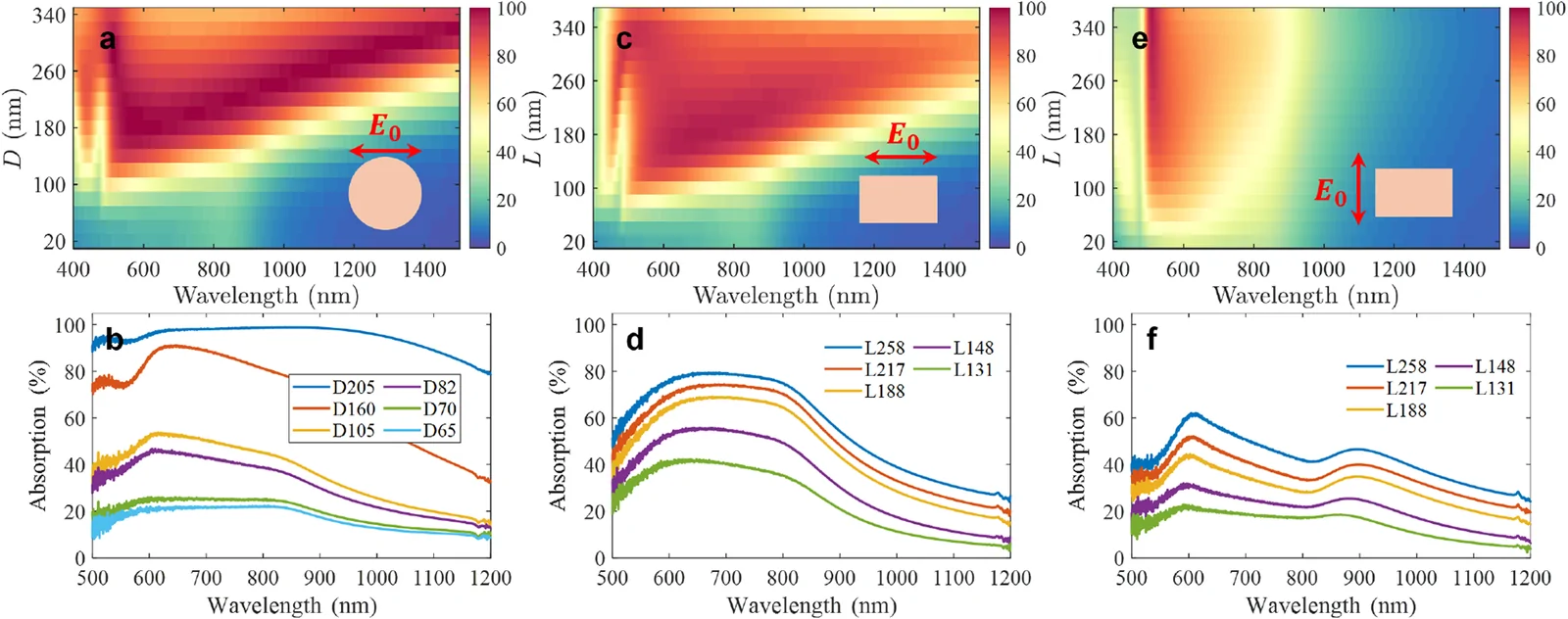
Fill-factor-controlled absorption in structured nanometer optical coatings. Simulated (**a**) and measured (**b**) absorption spectra of circular nanomagnet arrays with different diameters *D* under *x*-polarized incidence. Simulated (**c**) and measured (**d**) absorption spectra of rectangular nanomagnet arrays under *x*-polarized incidence, with *W* fixed and *L* varied. Simulated (**e**) and measured (**f**) absorption spectra of rectangular nanomagnet arrays under *y*-polarized incidence, with *W* fixed and *L* varied. Under *y*-polarized incidence, the electric field is parallel to the nanobar short axis *W*. Details of the geometric parameters are provided in Figure 3.

Figure 4c–f present the simulated and measured absorption spectra of the rectangular bar configuration for *x*-polarized incidence (Figure 4c,d) and *y*-polarized incidence (Figure 4e,f). For the rectangular arrays, the resonant absorption is influenced by the broad, geometry-dependent optical response of the lossy permalloy nanobars together with the Fabry–Pérot-type interference of the multilayer structure. Because this optical response is strongly loss-broadened, changes in magnetic-material fill factor substantially modify the effective amplitude of the metasurface reflection and therefore the coherent-absorption condition. For the best-performing geometry in this series, the experimental peak absorption approaches 80%, for a fill factor of 19.9%. In the case of incident polarization perpendicular to the bars, significant absorption may also occur, unless the bar width is reduced. Reducing the bar width decreases the fill factor and modifies the geometry-dependent optical response associated with the short axis of the bar.

It should be noted that the present work examines and tunes the light-nanomagnet interaction within a reduced parameter space, primarily through the Fabry–Pérot interference condition and the geometrical fill factor. Other nanomagnet parameters are kept fixed because they strongly influence the magnetic response, allowing the optical effects to be isolated. A more comprehensive optimization incorporating additional degrees of freedom, such as nanomagnet thickness, spacing, morphology, and material properties, may further enhance the interaction and could be explored in future work using inverse-design or machine-learning approaches.

### Enhanced MOKE in Ferromagnetic Nanobars

Through the design approach described, we have shown how to tune the interaction between light and a permalloy nanomagnet metasurface. In previous work, our motivation was to enhance all-optical switching of individual magnets within ASI.^7^ As a consequence of enhanced magneto-optical interaction, we can also expect to enhance the MOKE in these systems. MOKE is a sensitive technique that probes a material’s magnetization by analyzing the polarization change of reflected light upon interaction. While previous studies^18^ have used this concept to enhance polar MOKE signals in thin magnetic films, here we show that the enhancement is achievable for longitudinal MOKE in a metasurface of nanomagnets with in-plane magnetization.

Figures 5 and 6 present the results of MOKE measurements on each full array of permalloy rectangular bars of varying lengths. Because the nanomagnets are magnetized in-plane, longitudinal MOKE requires non-normal incidence. Here we use a NanoMOKE2 system in a longitudinal MOKE configuration with 633 nm wavelength light incident at 45 deg to the sample surface, as shown in Figure 5a. The laser spot size is slightly smaller than the size of each array, so the measured response is averaged over the array. Figure 5b shows the variation of the relative absorption as a function of bar nanomagnet length, evaluated by raster-scanning the illumination beam of the MOKE setup over the array, while recording the reflected intensity with the photodiode. The incident light had a wavelength of 633 nm and was linearly p-polarized. The relative absorption was inferred from the reflectance contrast between each patterned nanomagnet array and the adjacent bare cavity region (no permalloy). Since the bare cavity has a reflectivity below unity, this relative absorption is a lower bound on the absolute absorption of the array. We observe an increase in relative absorption as the magnet bar length increases to a maximum of 52%. The corresponding absorption remains lower than that observed under normal incidence for the arrays of Figure 4c and d; this reflects both the 45° angle of incidence and the smaller bar width (*W* = 80 nm) used for the MOKE series, which reduces the fill factor. It nevertheless remains substantial, demonstrating that strong light-matter interactions persist under oblique illumination. Additional FDTD simulations under the experimental 45° p-polarized geometry show reasonable agreement with the measured relative absorption and reproduce its increasing trend with nanobar length (Figure S4). The remaining quantitative differences are attributed mainly to deviations of the fabricated nanobars from the ideal rectangular geometry used in the simulations. The inset heatmap of Figure 5b further visualizes the variation in absorption from each of the measured magnetic metasurface arrays.

**Figure 5:**
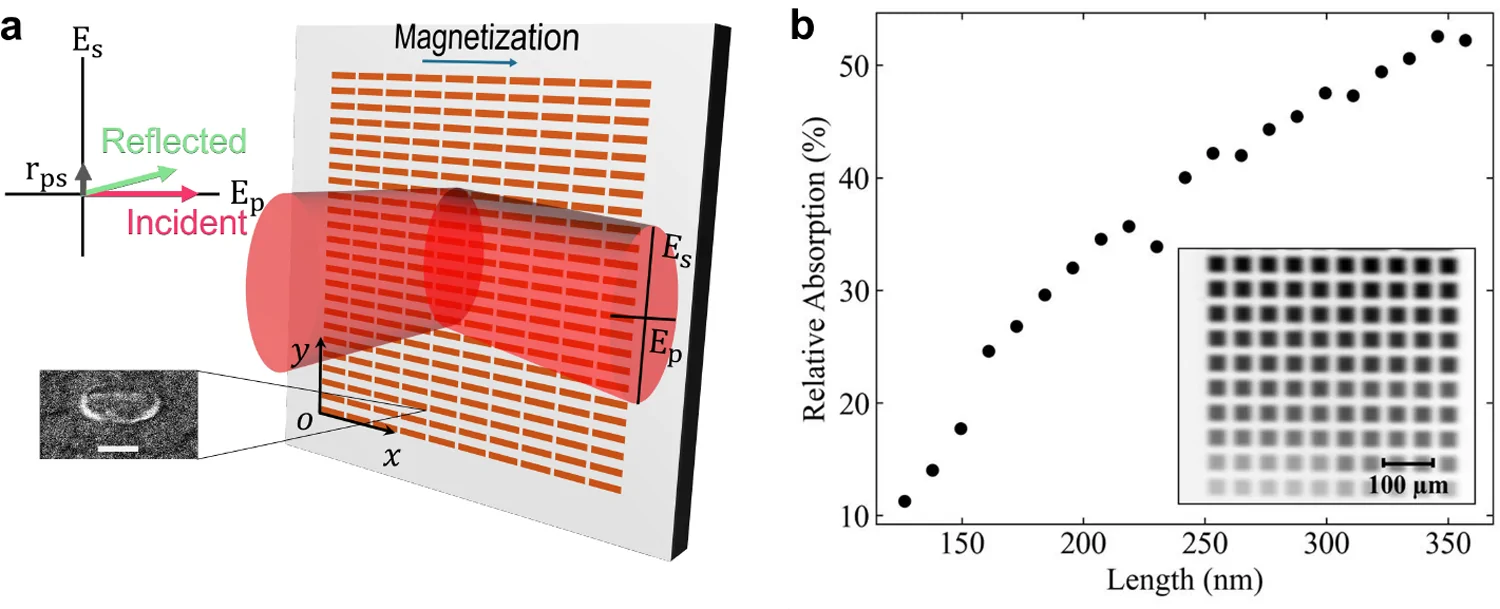
MOKE measurement configuration and relative absorption. (**a**) Diagram of the longitudinal MOKE geometry. The incidence angle is 45°. Inset: Representative SEM image of the fabricated permalloy nanobar. These are a separate set of samples from those in Figures 3 and 4, fabricated with the same four-layer cavity system as in Figure 3b and c but with the nanobar width fixed at *W* = 80 nm. Scale bar represents 100 nm. (**b**) Relative absorption (referenced to the bare cavity, see text) of 633 nm laser light for arrays with linearly increasing nanobar lengths, showing enhanced absorption with increasing length. Inset: Heatmap of absorption from scanning MOKE reflection measurements.

**Figure 6:**
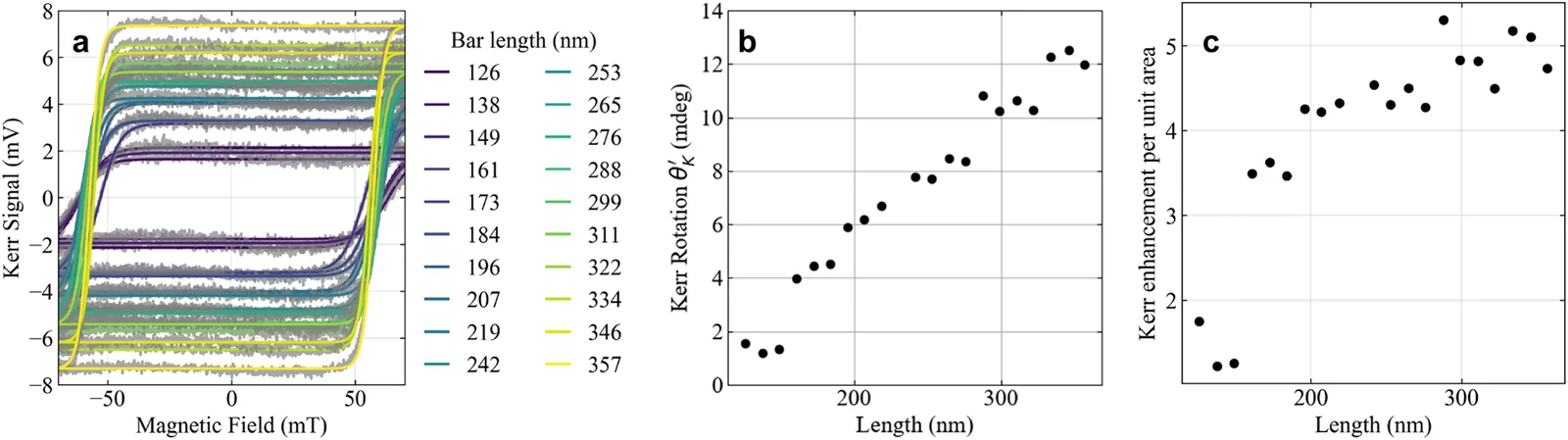
Results of the MOKE measurement. (**a**) Hysteresis loops of the MOKE signal for nanobars of fixed width *W* = 80 nm and lengths ranging from 126 to 357 nm, illustrating the length-dependent magneto-optical response. The color scale represents the nanobar length. (**b**) Absolute Kerr rotation angle (θ_*K*_^′^), obtained from the saturated MOKE signals and plotted as a function of nanobar length. The corresponding signal-to-noise ratio is reported in Figure S5. (**c**) Kerr rotation per magnetic-material area, normalized to the continuous 20 nm permalloy reference region on the same interference cavity. This area-normalized value is an efficiency metric and is distinct from the absolute Kerr rotation shown in panel **b**.

In longitudinal MOKE, p-polarized incident light is reflected with a small s-polarized component due to the magneto-optical Kerr effect, in addition to the dominant p-polarized component. The reflected electric field can be described as **E**_*r*_ = (*r*_*pp*_ **p̂** + *r*_*sp*_ **ŝ**)*E*_0_, where *r*_*pp*_ and *r*_*sp*_ are the nonmagneto-optical and magneto-optical reflection coefficients, respectively, while **p̂** and **ŝ** are polarization vectors. The intensity of light transmitted through an analyzer, set at a small offset angle θ_*a*_ from maximum extinction (2° in the current experiment), is given by *I*_*r*_ = *I*_0_|*r*_*pp*_ sin θ_*a*_ + *r*_*sp*_ cos θ_*a*_|^2^ ≈ *I*_*r*0_(1 + 2 θ_*K*_^′^/θ_*a*_), for |*r*_*sp*_| ≪ |*r*_*pp*_| and small θ_*a*_, where *I*_*r*0_ ≈ θ_*a*_^2^ |*r*_*pp*_|^2^ *I*_0_ is the reflected intensity without magnetic field and the Kerr rotation angle, θ_*K*_ = *r*_*sp*_/*r*_*pp*_ = θ_*K*_^′^ + *iθ*_*K*_^″^. Experimentally, we measure the intensity changes upon magnetization in forward (*I*_*M*_+__) and reverse (*I*_*M*_–__) directions to eliminate artifacts, where Δ*I* = *I*_*M*_+__ – *I*_*M*_–__ ≈ 4*I*_*r*0_θ_*K*_^′^/θ_*a*_.

Figure 6a shows the longitudinal MOKE hysteresis loops measured for nanobars with lengths ranging from 126 to 357 nm, with the different colors denoting the respective bar lengths. To quantify the length dependence of the MOKE response, the Kerr rotation angle at magnetic saturation was extracted from each hysteresis loop and plotted as a function of bar length in Figure 6b. Although the coercivity remains nearly independent of bar length, the magnitude of the normalized magneto-optical signal, Δ*I*/*I*_*r*0_, due to the Kerr polarization rotation, increases progressively with increasing bar length. The nonmagneto-optical signal, *I*_*r*0_, was background-corrected by subtracting the photodiode signal measured with the polarizer and analyzer in a crossed configuration, thereby accounting for contributions from the detector dark voltage and stray or scattered light. A signal-to-noise analysis of the measured hysteresis loops gives SNR values above 20 dB for all nanobar lengths (Figure S5), confirming that the observed length-dependent Kerr response is well resolved above the measurement noise. To provide a reference for evaluating the enhancement, we also measured a Kerr rotation of 12.5 mdeg from a continuous permalloy reference region on the same sample. This reference is a continuous 20 nm film deposited in the same evaporation step, on the same interference structure, and measured under identical conditions, so it differs from the arrays only in the absence of nanoscale patterning. Figure 6c shows the Kerr rotation enhancement normalized by the permalloy nanobar area. Importantly, the optical absorption and MOKE response were measured using the same series of nanomagnet arrays fabricated on the same Al back-plane interference structure. The nanobar length was varied to tune the absorption, while the substrate, multilayer cavity, magnetic material, and fabrication conditions remained unchanged. After normalization by the permalloy nanobar area, the Kerr response increases with nanobar length, reaching approximately five times the Kerr rotation per unit area of the continuous reference film for the longest bars (Figure 6c). This approximately 5-fold value is an area-normalized enhancement relative to the cavity-supported continuous-film reference and should not be interpreted as a 5-fold increase in the absolute Kerr rotation. Because the substrate, cavity, magnetic material, film thickness and measurement conditions are common to the entire series, this cannot be attributed simply to a larger amount of permalloy within the illuminated spot. This comparison shows that nanomagnets with stronger absorption generate a larger MOKE response per unit area of magnetic material.

The enhancement of MOKE polarization rotation, θ_*K*_ ≈ *r*_*sp*_/*r*_*pp*_, with bar length can be attributed to both an increased magneto-optical cross-reflection coefficient, *r*_*sp*_, and reduced specular reflection, *r*_*pp*_, in the interference design. Near-field coupling between adjacent nanostructures is expected to be weak in the present geometry because of the relatively large interstructure spacing. The enhanced MOKE is therefore primarily attributed to interference-enhanced absorption in the multilayer cavity system, while collective near-field coupling is expected to play a secondary role.

We note that the enhancement is expressed per unit area of magnetic material. In absolute terms the largest arrays reach a Kerr rotation comparable to that of the continuous reference film while covering only about a fifth of the surface, so the per-area figure is the appropriate measure of how efficiently light is coupled into the magnetic material. The absolute signal is bounded by the fill factor and could be raised further by denser arrays. We also note that the reference film sits on the same interference cavity as the arrays, so the quoted 5-fold enhancement is referenced to an already cavity-enhanced baseline and should not be interpreted as a 5-fold enhancement of the absolute Kerr rotation relative to bare permalloy.

It should be noted that the present MOKE measurements were performed at a fixed wavelength of 633 nm because the available magneto-optical setup employs a fixed-wavelength laser source, and wavelength-resolved Kerr spectra could therefore not be acquired. Importantly, 633 nm corresponds to the design wavelength of the interference structure. The MOKE measurement therefore directly probes the Kerr response under the interference-assisted absorption condition investigated in this work. In addition, the extracted Kerr rotation angles and enhancement factors should be regarded as semiquantitative estimates. The analysis assumes purely p-polarized incident light, an ideal linear analyzer, and fully coherent specular reflection, conditions that are not strictly realized because of optical imperfections and alignment uncertainties. Comparison with literature magneto-optical constants is further complicated by the sensitivity of patterned permalloy to fabrication conditions.

## Conclusions

We have shown that multilayer interference engineering offers an effective and robust strategy for dramatically enhancing optical absorption in nanoscale magnetic metasurfaces. By integrating permalloy nanomagnet arrays with a thin dielectric spacer and a reflective metallic back-plane, we achieve a metasurface environment in which geometric parameters—including nanomagnet shape, fill factor, and cavity thickness—determine the interference conditions for incident light. Tuning these parameters allows the system to reach destructive interference in reflection, enabling near-unity absorption concentrated within the magnetic nanostructures.

This absorption enhancement is accompanied by stronger MOKE signals, driven by both increased light-nanomagnet interaction and reduced reflected background. The 500–1200 nm range investigated here follows from the interference condition designed at an operating wavelength of 633 nm, together with the optical properties of the constituent materials and the response range of our FTIR setup. Importantly, the underlying mechanism is general and can be extended to other wavelength regimes through appropriate material selection and geometric scaling. The demonstrated strategy addresses the limited optical interaction volume of ultrathin magnetic nanostructures and provides a pathway toward compact and efficient opto-magnetic platforms. More broadly, our approach is applicable to a wide range of photonic, plasmonic, and metasurface-based structures where strong absorption in ultrathin functional layers is desired. These findings open new directions in optically driven magnetism, magnetic metasurface engineering, and light-enabled spintronic technologies.

## Methods

### Optical Simulation Details

Simulations of the linear optical response of the nanostructures were performed using the finite-difference time-domain (FDTD) method. Linearly polarized plane waves of varying wavelength and polarization were injected from the air side onto the periodic array; the polarization used in each case is indicated in the corresponding figure. Periodic boundary conditions were applied in the *x*- and *y*-directions, and perfectly matched layers were used in the *z*-direction to absorb outgoing light with minimal back-reflection. The optical constants used in the FDTD simulations were taken from experimentally measured wavelength-dependent data sets. The optical constants of permalloy were taken from ref 47, Au from the Johnson and Christy data set,^48^ and SiO_2_, Al_2_O_3_, Si, and Al from Palik.^49^ The corresponding complex dielectric functions are plotted in the Supporting Information (Figure S3). The wavelength-dependent optical properties of these materials determine the interference, absorption, and field confinement characteristics of the metasurface system within the investigated spectral range. For the ferromagnetic permalloy, although the dielectric function becomes tensorial in the magnetized state, only the diagonal complex permittivity was used in the FDTD simulations, as the off-diagonal terms are much smaller and are expected to have a negligible effect on the absorption features considered here, consistent with the measurements in Supporting Information Figure S2.

### Experimental Methods

Samples were fabricated via electron-beam lithography and thermal evaporation. Two back-plane variants were used: Au and Al. For the Au back-plane, a Cr­(2 nm)/Au­(250 nm)/Cr­(2 nm) stack was thermally evaporated onto a silicon wafer, the Cr layers improving adhesion of Au to the substrate and of the silica to the Au. For the Al back-plane, a 250 nm Al film was thermally evaporated onto a silicon wafer, thick enough that transmission is negligible across 500–1200 nm. A SiO_2_ spacer of the stated nominal thickness was then deposited by sputtering in an Angstrom Engineering AMOD system, with the thickness confirmed by ellipsometry. All samples reported here use SiO_2_; Al_2_O_3_ is included in Figure 1a as an equivalent low-loss alternative for the spacer layer. Then, the substrate was spin-coated with a bilayer 495*K*/950 K poly­(methyl methacrylate) (PMMA) resist layer and baked at 180 °C for 5 min. The nanostructures were subsequently defined by electron-beam exposure, followed by development in a standard 1:3 methyl isobutyl ketone/isopropanol (MIBK/IPA) solution (for 40 s). A 20 nm permalloy (Ni_81_Fe_19_) film was thermally evaporated at a base pressure of 2 × 10^–6^ mbar, and the lift-off process was performed in acetone as a final step.

### MOKE Experiments

The longitudinal MOKE measurements were performed using a Durham Magneto-Optics NanoMOKE2 system at room temperature. A 633 nm diode laser was focused to a ∼ 20 μm spot diameter onto the nanomagnet array and an external magnetic field was applied in-plane, parallel to the long axis of the magnetic bars. To compare the different array dimensions the incident laser polarization was kept constant manually at an offset of 2° from the extinction angle defined by the polarizer-analyzer alignment.

Hysteresis loops were measured under a quasistatic sinusoidal magnetic field generated by a quadrupole electromagnet operating at 10 Hz. Signals were averaged over 1000 field cycles. The MOKE signal was normalized by linear background subtraction.

### Fourier Transform Infrared Measurements

FTIR spectroscopy was used to characterize the linear optical response of the fabricated nanomagnet arrays. Spectra were collected with a Bruker Hyperion 2000 FTIR microscope equipped with a 36×, NA = 0.5 reflective objective. Reflection spectra were obtained by normalizing the signal from the patterned array region to a reference spectrum recorded from a silver mirror located close to the array on the same sample. Measurements were made over 500–1100 nm and 1100–1200 nm using a silicon detector and a Peltier-cooled InGaAs detector, respectively.

## Supplementary Material



## Notes

Comparison with established magneto-optical Kerr effect enhancement strategies; simulated absorption contribution of the metallic back-plane; effect of the magnetic field on optical reflectance and absorption spectra; optical constants of the constituent materials; FDTD simulations under longitudinal-MOKE measurement conditions; signal-to-noise ratio analysis of MOKE hysteresis measurements; and comparison of representative MOKE enhancement approaches (PDF)
