# Supplementary material for: Enhanced Magneto-Optical Kerr Effect in Nano-Magnetic Metamaterials with Near-Perfect Coherent Absorption
Source: ACS Nano. 2026 Oct 21;20(43):29188–97. doi: 10.1021/acsnano.6c15462 (PMC13738692; doi:10.1021/acsnano.6c15462)
Supplement: Supplementary file 1 [file nn6c15462_si_001.pdf]

Supporting Information: Enhanced Magneto-Optical Kerr Effect in Nano-Magnetic Metamaterials with Near-Perfect Coherent Absorption

Xiaofei Xiao,[*,†,‡] Tingjun Zheng,[†,‡] Alex Vanstone,[†,‡] Daniel S. Bromley,[†] Holly Holder,[†] Kilian D. Stenning,[†] Jack C. Gartside,[†] Will R. Branford,[†] and Rupert F. Oulton[*,†]

†*Blackett Laboratory, Imperial College London, London SW7 2AZ, United Kingdom*

‡*Contributed equally to this work*

E-mail: xiaofei.xiao15@imperial.ac.uk; r.oulton@imperial.ac.uk

1. Comparison with Established Kerr-Enhancement Strategies

To place the present work in context, we compare our approach with representative magneto-optical Kerr effect (MOKE) enhancement strategies reported in the literature, as summarized in Table S1. Existing approaches can be broadly categorized into:

1. interference-based cavities,
2. plasmonic/magneto-plasmonic structures,
3. dielectric resonant metasurfaces and photonic resonators,

4. intrinsic/interface material engineering approaches,
5. measurement-side amplification techniques.

Interference-based cavity enhancement

Interference-assisted approaches, including Fabry–Pérot cavities and multilayer antireflection structures, enhance magneto-optical responses through controlled optical interference within or around the magnetic layer.[S1] Such approaches have been demonstrated in continuous magnetic films. For example, Kim et al.[S2] used an extreme-antireflection structure to strongly suppress the non-magneto-optical reflected background and enhance the Kerr response of an ultrathin continuous magnetic film. These studies demonstrate the effectiveness of interference engineering for magnetic thin films; however, the magnetic layer generally remains laterally continuous, and its magnetic geometry is not itself used as a primary degree of freedom for establishing the optical interference condition.

Plasmonic and magneto-plasmonic enhancement

Plasmonic and magneto-plasmonic systems rely on localized or propagating plasmon resonances to strongly concentrate electromagnetic fields near magnetic nanostructures.[S3–S6] Such near-field enhancement can substantially increase light-matter interaction and produce large Kerr responses. Representative examples include noble-metal/ferromagnetic hybrid nanostructures and magneto-plasmonic metasurfaces. However, plasmonic enhancement is commonly accompanied by significant Ohmic losses, thermal dissipation, and broader resonance linewidths. In addition, the integration of noble-metal nanostructures generally increases fabrication complexity. A recent example of this trade-off is the numerically designed resonator of Wang et al.,[S6] in which hexagonally arrayed Au/bismuth-iron-garnet (BIG) bilayer nanodisks on an Au film couple grating-excited surface plasmons to Au–BIG–Au waveguide modes. In this system, the magneto-optically active BIG layer is optically trans-

parent, and the enhanced transverse MOKE is associated with strong plasmon–waveguide mode coupling and near-field interference.

Dielectric resonant metasurfaces and photonic resonators

All-dielectric metasurfaces and photonic resonators enhance magneto-optical interactions through low-loss Mie resonances, guided resonances, or quasi-bound states in the continuum (quasi-BICs).[S7,S8] Compared with plasmonic systems, dielectric resonators can support high-Q optical confinement with reduced dissipative loss. However, preserving high-Q resonant behavior typically requires tighter dimensional tolerances and increased sensitivity to geometry, polarization, and incidence conditions.

Intrinsic and interface material engineering

Another class of enhancement strategies focuses on improving the intrinsic magneto-optical properties of the material system itself, for example through interface hybridization, magnetic multilayer engineering, strain engineering, or spin-orbit coupling enhancement.[S9] These approaches can enhance Kerr signals without relying on photonic resonances or nanostructured optical confinement, although their tunability is generally more limited compared with resonant photonic platforms.

Measurement-side amplification approaches

Measurement-side enhancement techniques, such as weak-value amplification and interferometric detection schemes, improve the detectability of Kerr rotation through post-selection and optical amplification methods.[S10] While these approaches can significantly increase measurement sensitivity, they do not directly enhance the intrinsic light-magnetization interaction inside the material system.

Present work: interference-assisted absorption in patterned magnetic nanostructures

The present work extends interference-assisted absorption engineering to lithographically patterned metallic nanomagnets. Here, the nanomagnet shape, dimensions, and magnetic-material fill factor determine the effective reflection and transmission coefficients of the metasurface and therefore participate directly in establishing the coherent-absorption condition, while the same geometrical parameters also define the magnetic functionality of the nanostructures. The key distinction from conventional interference-enhanced continuous magnetic films is therefore not the use of thin-film interference itself, but the use of the patterned magnetic geometry as an active optical design parameter.

Under near-perfect absorption conditions, optical energy is efficiently coupled and dissipated within the magneto-optically active permalloy nanomagnets, leading to enhanced light–nanomagnet interaction and stronger MOKE responses. The system therefore combines Fabry–Pérot-type interference with the geometry-dependent optical response of a functional magnetic metasurface.

Compared with plasmonic approaches, the present strategy does not rely on extreme plasmonic hot-spot confinement as the dominant enhancement mechanism. The contrast with Ref. S6 is instructive: there the magneto-optically active layer is transparent and the enhancement arises from plasmon–waveguide mode coupling and near-field interference, whereas here the active magnetic layer is itself the absorber and the incident energy is routed into it through interference-assisted suppression of reflection. Ref. S6 treats the transverse Kerr effect numerically, while the enhancement reported here is measured in the longitudinal configuration. Compared with ultra-high-Q dielectric resonators, the demonstrated system does not require extremely narrow resonance linewidths to achieve significant Kerr enhancement. Instead, the mechanism combines Fabry–Pérot-type interference with the geometry-dependent optical response of the magnetic metasurface, providing a compact and lithographically tunable platform compatible with metallic ferromagnetic nanostructures.

The primary trade-offs of the present approach are that the response remains dependent on geometry and interference conditions, while the strong absorption condition is accompanied by reduced reflected intensity near resonance. Nevertheless, this strategy provides a practical pathway for enhancing magneto-optical responses through interference-assisted absorption engineering and may be extended to a broad range of photonic, plasmonic, and metasurface-based systems where strong optical absorption within ultra-thin functional layers is desirable.

2. Absorption Contribution of the Metal Mirror

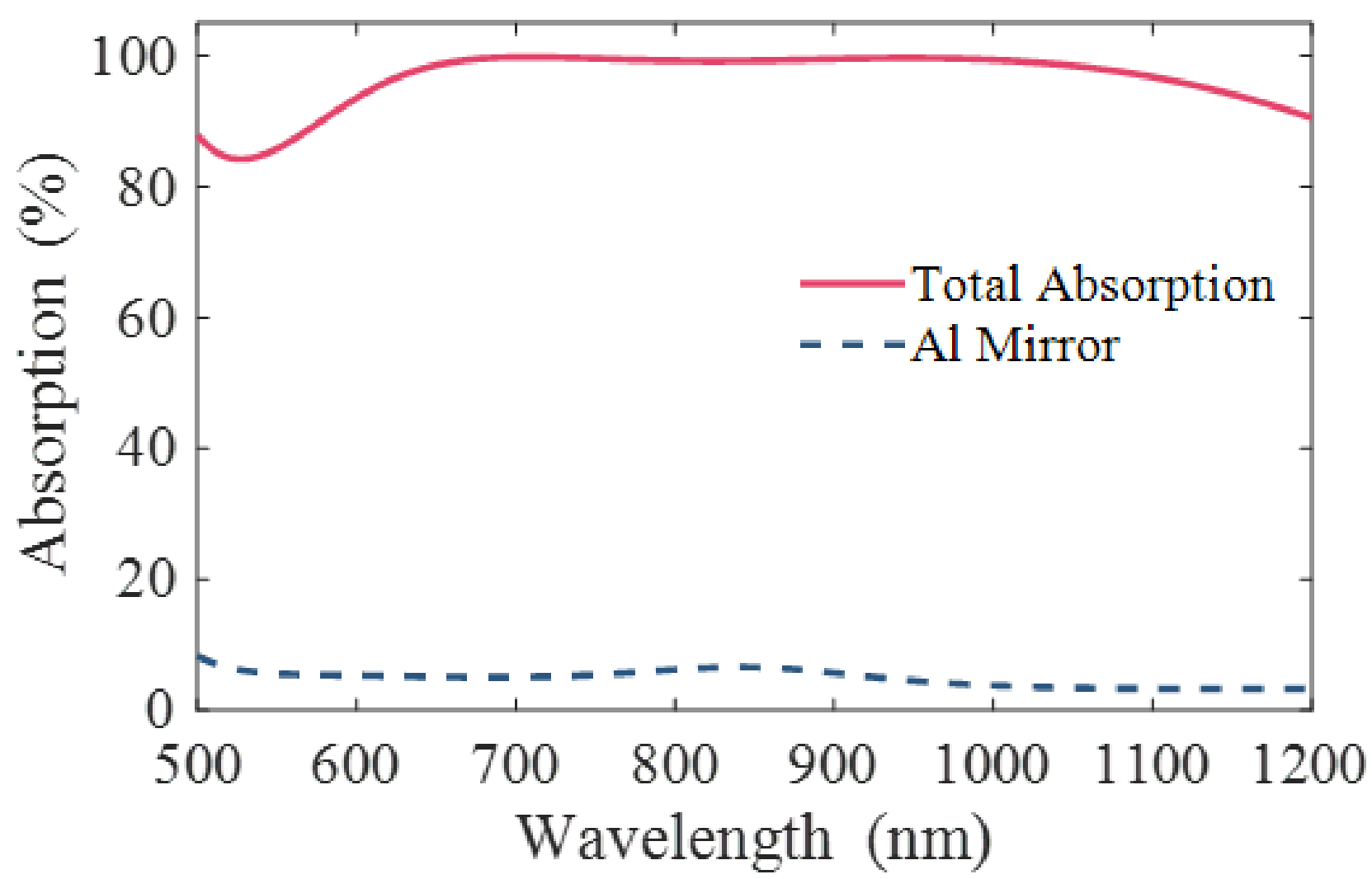

Figure S1: Simulated absorption spectra of the nanomagnet metasurface and the Al mirror within that same structure, for a SiO_2 spacer thickness of 92 nm. The designed nanomagnet system has geometrical parameters of $l_1 = 20$ nm, $L = 250$ nm, $W = 110$ nm, $d = 250$ nm, and $P = 360$ nm. The schematic of the nanomagnet array structures is the same as that shown in Figure 2, except that the mirror is Al rather than Au.

Figure S1 compares the total absorption of the combined system with the absorption contribution from the Al mirror within the same system. The absorption associated with the Al mirror remains relatively low (typically below ∼5%) across the visible-near-infrared spectral range, whereas the complete metasurface structure achieves near-unity absorption over a

broad wavelength range. These results confirm that the enhanced absorption predominantly originates from the engineered impedance-matching condition and interference-assisted light trapping enabled by the metasurface cavity structure, rather than from absorption in the Al mirror itself.

3. Effect of the Magnetic Field on the Optical Spectrum

Additional Fourier-transform infrared (FTIR) spectroscopy measurements were performed to investigate whether the magnetization state measurably affects the optical response of the interference structure. The measured sample consisted of a 20-nm-thick thermally deposited permalloy film on a 92 nm SiO_2 spacer and an optically opaque Al back-plane. Reflectance spectra were recorded in the demagnetized state and under positive and negative magnetic saturation. All spectra were normalized to a silver-mirror reference, whose reflectivity is close to unity over the measured spectral range.

As shown in Fig. S2, the reflectance spectra measured in the three magnetic states overlap within the experimental uncertainty. This indicates that the magnetization state of the permalloy film produces no resolvable change in the broadband reflectance spectrum under the present measurement conditions. Because the Al back-plane suppresses transmission through the structure, the absorption can be obtained from $A = 1 - R$. The corresponding absorption spectra therefore also overlap within the experimental uncertainty, showing that neither the applied magnetic field nor the direction of magnetization measurably modifies the broadband absorption spectrum.

This result is consistent with the magneto-optical response being much smaller than the dominant optical response of the multilayer structure. The strong absorption is governed primarily by the diagonal optical response through resonant field enhancement and Fabry–Pérot-type interference, whereas magnetization introduces much smaller off-diagonal terms. These off-diagonal terms are more sensitively detected through changes in the polarization

state of the reflected light in the MOKE measurement.

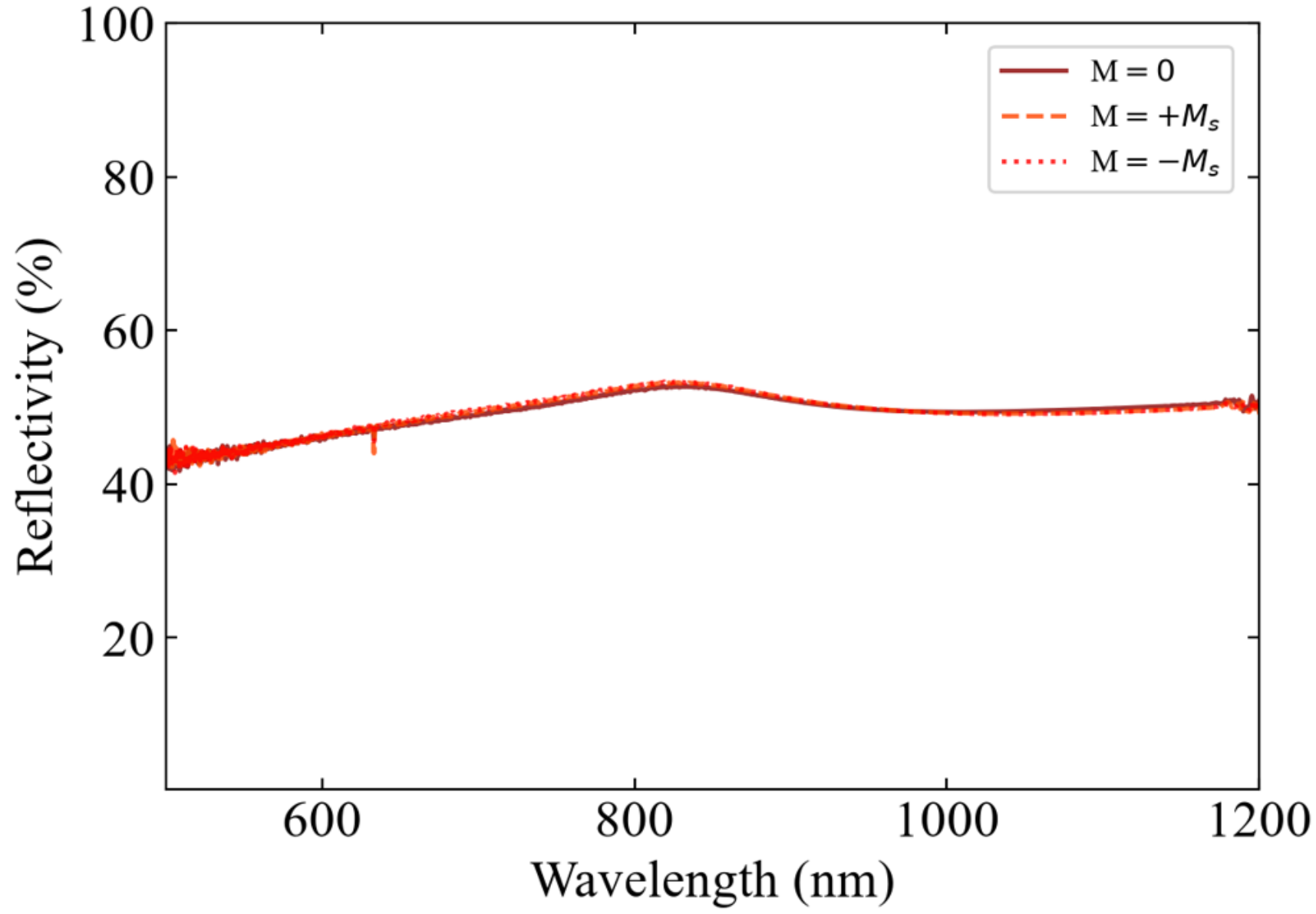

Figure S2: FTIR reflectivity spectra of a 20-nm-thick permalloy film deposited on the Al back-plane interference structure, measured in the demagnetized state ($M = 0$) and under positive ($M = +M_{\mathrm{s}}$) and negative ($M = -M_{\mathrm{s}}$) magnetic saturation.

4. Optical Constants of the Constituent Materials

Figure S3 shows the complex dielectric functions of all materials used in the FDTD simulations, over the spectral range relevant to this work. The data sources are given in the Methods section of the main text. These data account for the spectral window used here: the spacers are transparent and dispersionless, the ϵ'' of permalloy increases monotonically towards the infrared, and Au shows interband absorption below ∼515 nm while Al retains reflectivity further into the blue, with parasitic absorption below ∼5% over most of the band (Figure S1).

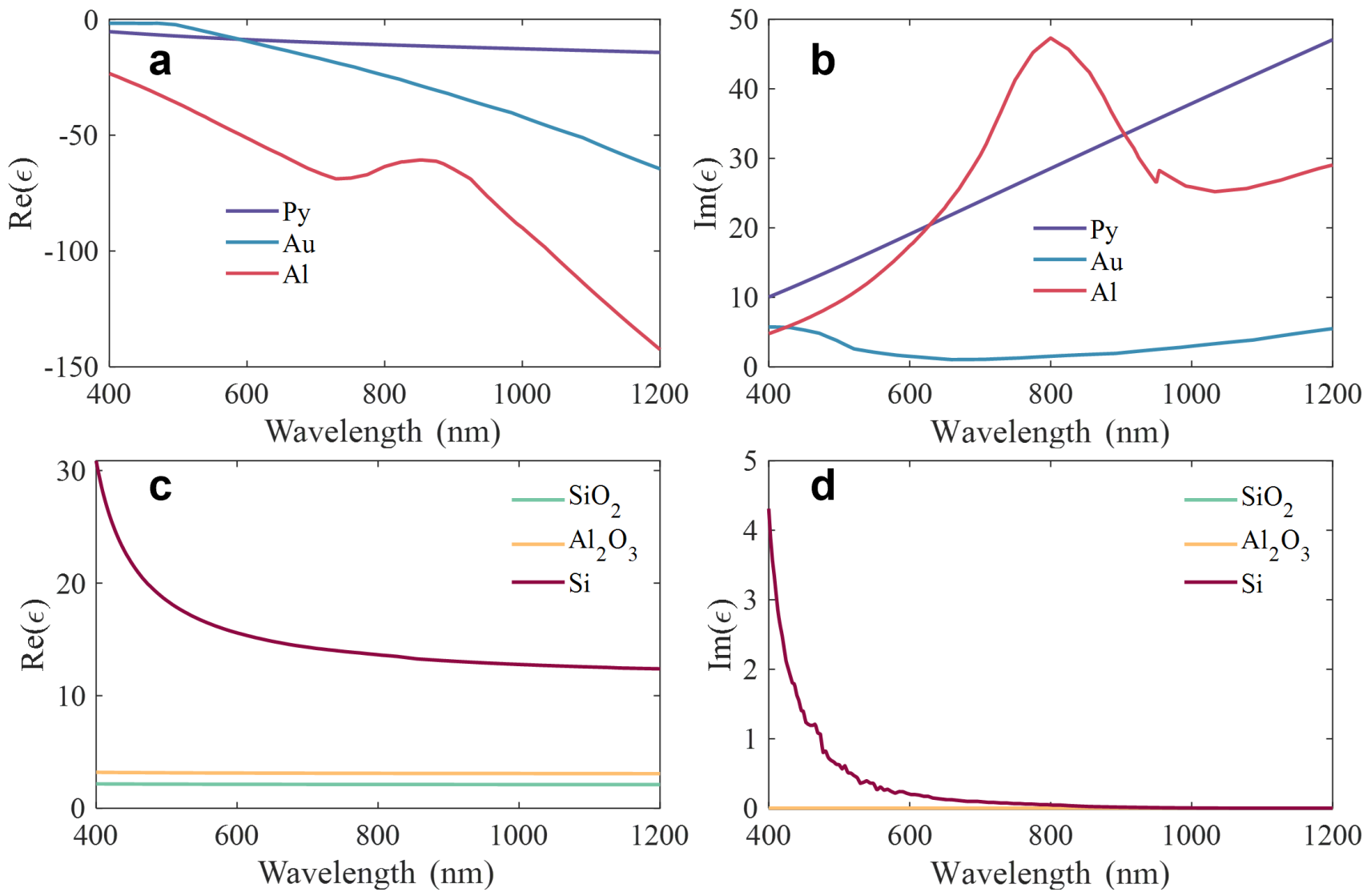

Figure S3: Complex dielectric function $\epsilon = \epsilon' + i\epsilon''$ of the materials used in the FDTD simulations. (**a**) Real and (**b**) imaginary parts for the metallic constituents (permalloy, Au, Al). (**c**) Real and (**d**) imaginary parts for the dielectric constituents (SiO_2, Al_2O_3, Si). Note the different vertical scales between the upper and lower rows; in (**c**) and (**d**) the SiO_2 and Al_2O_3 curves are nearly constant at $\epsilon' \simeq 2.1$ and 3.1 with $\epsilon'' \simeq 0$. Data sources are given in the Methods section of the main text.

5. FDTD simulation under the longitudinal-MOKE measurement geometry

To reproduce the longitudinal-MOKE measurement conditions, additional FDTD simulations were performed using p-polarized light incident at 45° with a wavelength of 633 nm. The nanobar width was fixed at $W = 80$ nm, while the nanobar length was varied. The simulated relative absorption was calculated as $A_{\text{rel}} = 1 - R_{\text{array}}/R_{\text{cavity}}$, consistent with the experimental analysis.

As shown in Figure S4, the simulated relative absorption increases with nanobar length, reproducing the experimentally observed trend. Comparison with the measured values in Figure 5b shows reasonable agreement. The remaining quantitative differences are attributed mainly to deviations of the fabricated nanobars from the ideal rectangular geometry used in the simulations.

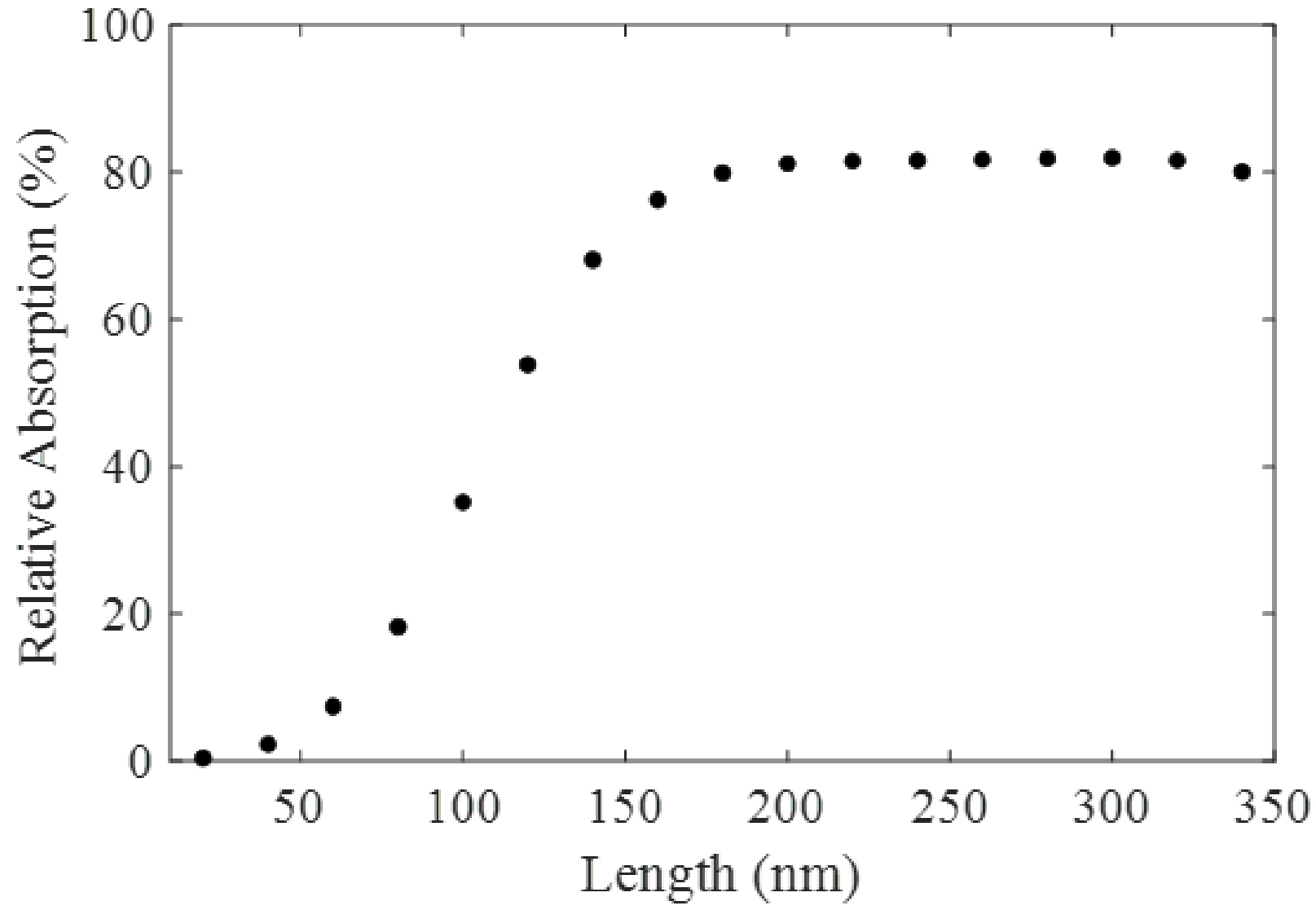

Figure S4: **FDTD simulation under the longitudinal-MOKE measurement geometry.** Simulated relative absorption at 633 nm as a function of nanobar length for arrays with fixed width $W = 80$ nm under p-polarized light incident at 45°. The simulated relative absorption is calculated as $A_{\text{rel}} = 1 - R_{\text{array}}/R_{\text{cavity}}$, consistent with the experimental normalization.

6. Signal-to-noise ratio of the MOKE hysteresis measurements

The signal-to-noise ratio (SNR) of the MOKE measurements was evaluated using the fitted Kerr signal and the residual fluctuations within the saturated regions of the hysteresis loops. Restricting the noise analysis to these regions reduces contributions from switching variability, field uncertainty, and fitting mismatch near the coercive fields. Each hysteresis branch was described by a Boltzmann bent-step function,

$$f(B) = y_0 + \frac{h}{2} \tanh\left(\frac{B - B_0}{w}\right) + aB + bB^2, \tag{1}$$

where B is the applied magnetic field, B_0 is the coercive-field parameter, and w controls the switching width. The parameter y_0 describes the signal offset, while a and b account for residual field-dependent background in the SNR fitting procedure. The full step height

of the hyperbolic-tangent component defines the background-corrected Kerr signal, $\Delta I = I_{M+} - I_{M-} = |h|$, where I is the photodetector signal measured in mV.

Noise was estimated within selected saturated-field regions away from the switching transitions, where the magnetization remains approximately constant. Residuals were calculated by subtracting the fitted response from the measured signal at the corresponding field values, $r = I(B) - f(B)$, and their standard deviation, σ_r, was used to estimate the noise.

The SNR was defined as the ratio of the fitted Kerr signal to the residual noise and expressed in decibels:

$$\mathrm{SNR}_{\mathrm{dB}} = 20 \log_{10}\left(\frac{|h|}{\sigma_r}\right). \tag{2}$$

The factor of 20 is used because the ratio compares signal amplitudes rather than powers. As shown in Figure S5, all resulting SNR values are above 20 dB, confirming that the length-dependent Kerr response is experimentally resolved above the measurement noise.

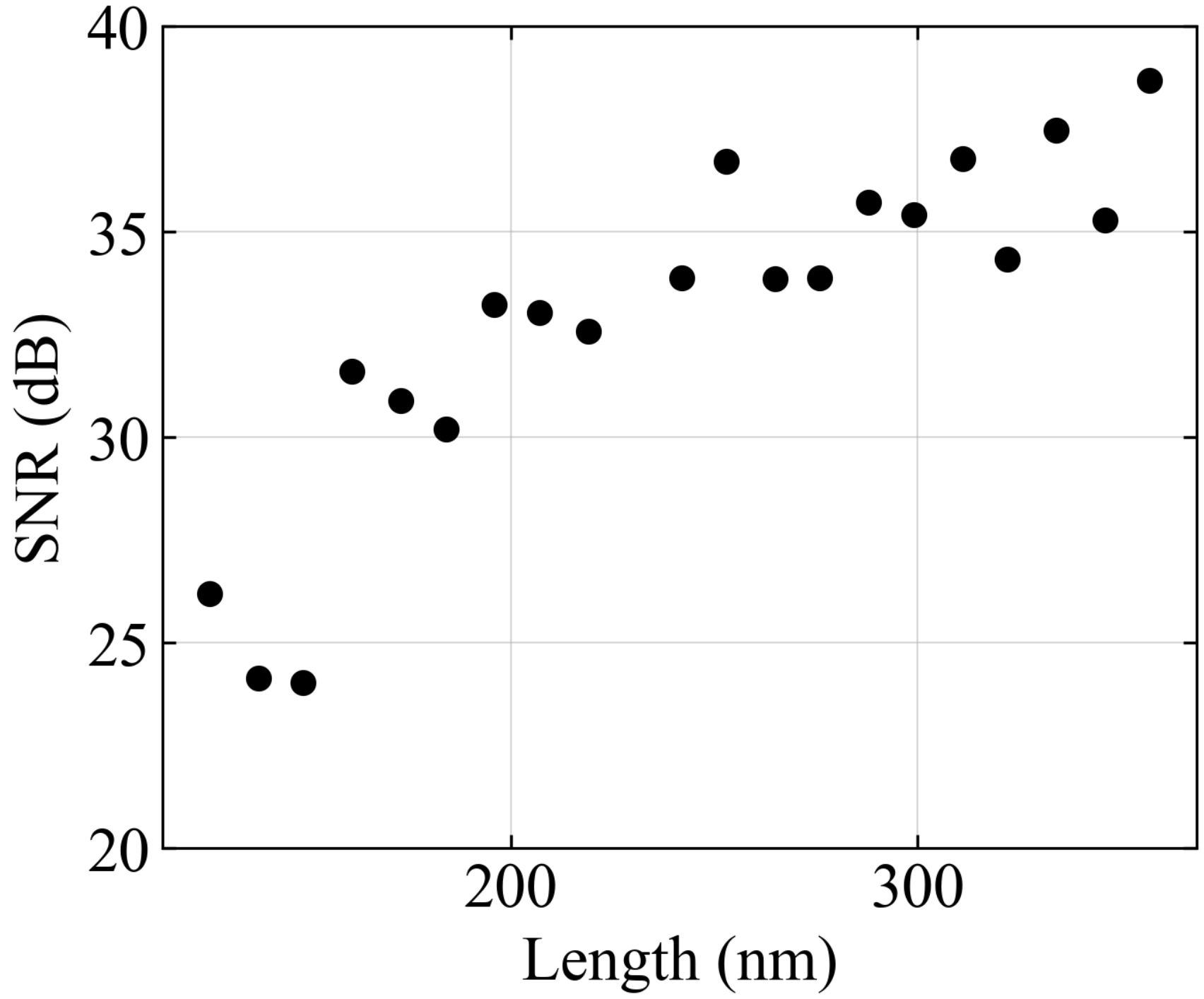

Figure S5: **Signal-to-noise ratio of the longitudinal MOKE measurements.** SNR extracted from the measured hysteresis loops as a function of nanobar length. The signal is defined as the absolute fitted full step height of the hysteresis response, while the noise is obtained from the standard deviation of the fit residuals within the saturated-field regions. All measured arrays exhibit SNR values above 20 dB.

Table S1: Comparison of representative MOKE enhancement strategies.

**Technique**	**Main mechanism**	**Strengths**	**Typical trade-offs**	**Representative refs**
Fabry–Pérot / interference cavities	Multiple reflections and interference enhance interaction in magnetic layer	Structurally simple; compatible with planar multilayers	Typically demonstrated with laterally continuous magnetic films; thickness- and phase-sensitive	S1,S2
Plasmonic / magneto-plasmonic structures	Localized plasmonic near-field enhancement	Strong local field enhancement; large Kerr response	Ohmic loss; thermal dissipation; fabrication complexity	S3–S6
Dielectric metasurfaces / photonic resonators	High-Q optical confinement via Mie / guided / quasi-BIC resonances	Low dissipative loss; strong spectral selectivity	Sensitive to geometry, polarization, and angle	S7,S8
Intrinsic / interface material engineering	Modification of intrinsic magneto-optical properties	No photonic structure required	Limited tunability	S9
Weak-value amplification	Measurement-side amplification of Kerr rotation	High detection sensitivity	Does not enhance intrinsic interaction	S10
This work	Interference-assisted coherent absorption with patterned magnetic geometry controlling metasurface reflection and transmission	Magnetic geometry jointly defines optical response and magnetic functionality; compatible with metallic nanomagnets	Geometry- and interference-dependent; reduced reflected intensity near resonance	This work

References

(S1) Nývlt, M.; Ferré, J.; Jamet, J. P.; Houdy, P.; Boher, P.; Višňovský, Š.; Urban, R.; Lopušník, R. Magneto-optical effects in a stack of magnetic multilayer-dielectric films. *Journal of Magnetism and Magnetic Materials* **1996**, *156*, 175–176.

(S2) Kim, D.; Oh, Y.-W.; Kim, J. U.; Lee, S.; Baucour, A.; Shin, J.; Kim, K.-J.; Park, B.-G.; Seo, M.-K. Extreme anti-reflection enhanced magneto-optic Kerr effect microscopy. *Nature communications* **2020**, *11*, 5937.

(S3) Belotelov, V.; Akimov, I.; Pohl, M.; Kotov, V.; Kasture, S.; Vengurlekar, A.; Gopal, A. V.; Yakovlev, D.; Zvezdin, A.; Bayer, M. Enhanced magneto-optical effects in magnetoplasmonic crystals. *Nature nanotechnology* **2011**, *6*, 370–376.

(S4) Armelles, G.; Cebollada, A.; García-Martín, A.; González, M. U. Magnetoplasmonics: combining magnetic and plasmonic functionalities. *Advanced Optical Materials* **2013**, *1*, 10–35.

(S5) López-Ortega, A.; Zapata-Herrera, M.; Maccaferri, N.; Pancaldi, M.; Garcia, M.; Chuvilin, A.; Vavassori, P. Enhanced magnetic modulation of light polarization exploiting hybridization with multipolar dark plasmons in magnetoplasmonic nanocavities. *Light: Science & Applications* **2020**, *9*, 49.

(S6) Wang, Z.; Cui, X.; Song, Y. Magnetoplasmonic Resonators Designed with Hexagonally Arrayed Au/BIG Bilayer Nanodisks on Au Thin Film Layers for Enhanced MOKE and Refractive Index Sensing. *Coatings* **2025**, *15*, 601.

(S7) Barsukova, M. G.; Shorokhov, A. S.; Musorin, A. I.; Neshev, D. N.; Kivshar, Y. S.; Fedyanin, A. A. Magneto-optical response enhanced by Mie resonances in nanoantennas. *Acs Photonics* **2017**, *4*, 2390–2395.

(S8) Gao, S.; Ota, Y.; Tian, F.; Liu, T.; Iwamoto, S. Optimizing the optical and magneto-optical response of all-dielectric metasurfaces with tilted side walls. *Optics Express* **2023**, *31*, 13672–13682.

(S9) Gu, B.; Takahashi, S.; Maekawa, S. Enhanced magneto-optical Kerr effect at Fe/insulator interfaces. *Physical Review B* **2017**, *96*, 214423.

(S10) Huang, J.-H.; Lundeen, J. S.; Jordan, K. M.; Dada, A. C.; Wang, G.-J.; Hu, X.-Y. Weak-value-amplification enhancement of the magneto-optical Kerr effect in nanoscale layered structures. *Physical Review A* **2023**, *108*, 033724.
